# A Case of Immunotherapy Response to BRAF^V600E^ ‐Mutant Lung Adenocarcinoma With Initial Resistance to Dabrafenib and Trametinib Combination Therapy

**DOI:** 10.1002/cnr2.70533

**Published:** 2026-03-31

**Authors:** Yusuke Okayama, Takaaki Tokito, Shizuka Shiraishi, Reiko Takaki, Shingo Tsuneyoshi, Hiroyoshi Yamada, Mamoru Nishiyama, Yoshiko Sueyasu, Tomoaki Hoshino

**Affiliations:** ^1^ Department of Respiratory Medicine Saiseikai Futsukaichi Hospital Chikushino Fukuoka Japan; ^2^ Division of Respirology, Neurology, and Rheumatology Kurume University Hospital Kurume Fukuoka Japan

**Keywords:** BRAF V600E, dabrafenib, immune checkpoint inhibitor, PD‐L1, trametinib

## Abstract

**Introduction:**

The recommended first‐line therapy for BRAF V600E mutant non‐small cell lung cancer (NSCLC) is a combination of dabrafenib and trametinib. Most patients respond to the initial therapy, but some show resistance in the early stages. Additionally, immune checkpoint inhibitors (ICIs) are often used after resistance develops; however, the benefits of ICIs in patients with BRAF V600E mutant NSCLC remain unclear.

**Case Presentation:**

We report a case of a 67‐year‐old man who was clinically diagnosed with lung adenocarcinoma cT2aN3M1c (BRA) stage IVB (BRAF V600E mutation‐positive, programmed death‐ligand 1; 90%). The patient developed early resistance to the combination of dabrafenib and trametinib but responded well to ICI.

**Conclusion:**

This case suggests that ICI may provide durable clinical benefit even after early resistance to BRAF/MEK inhibition in selected patients with high PD‐L1 expression.

## 1 Introduction

The development of personalized treatment strategies has led to significant improvements in the survival rates of patients with non‐small cell lung cancer (NSCLC). The BRAF V600E mutation is relatively rare, accounting for approximately 1%–3% of all NSCLC [1]. The prognostic impact of BRAF V600E mutations remains unclear; however, platinum‐based chemotherapies are less effective and have been reported to have a poor prognosis [2]. A phase II study of patients with unresectable advanced or recurrent NSCLC with the BRAF V600E mutation demonstrated that a combination of dabrafenib and trametinib obtained 64% of the overall response rate (ORR) and 10.8 months as median progression‐free survival by inhibiting cell growth signals at two sites of action in the mitogen‐activated protein kinase (MAPK) signaling pathway [3].

In contrast, PD‐1/PD‐L1 (programmed death‐ligand 1) immunotherapy can effectively improve prognosis. However, its therapeutic efficacy is poor in NSCLC with epidermal growth factor receptor (EGFR) and anaplastic lymphoma kinase (ALK) mutations [4]. The benefits of immune checkpoint inhibitors (ICIs) to patients with NSCLC having BRAF mutations are unknown. Here, we report a case in which immunotherapy was effective in a patient with BRAF V600E mutant NSCLC who initially showed resistance to combination therapy with dabrafenib and trametinib.

## 2 Case Presentation

**FIGURE 1:**
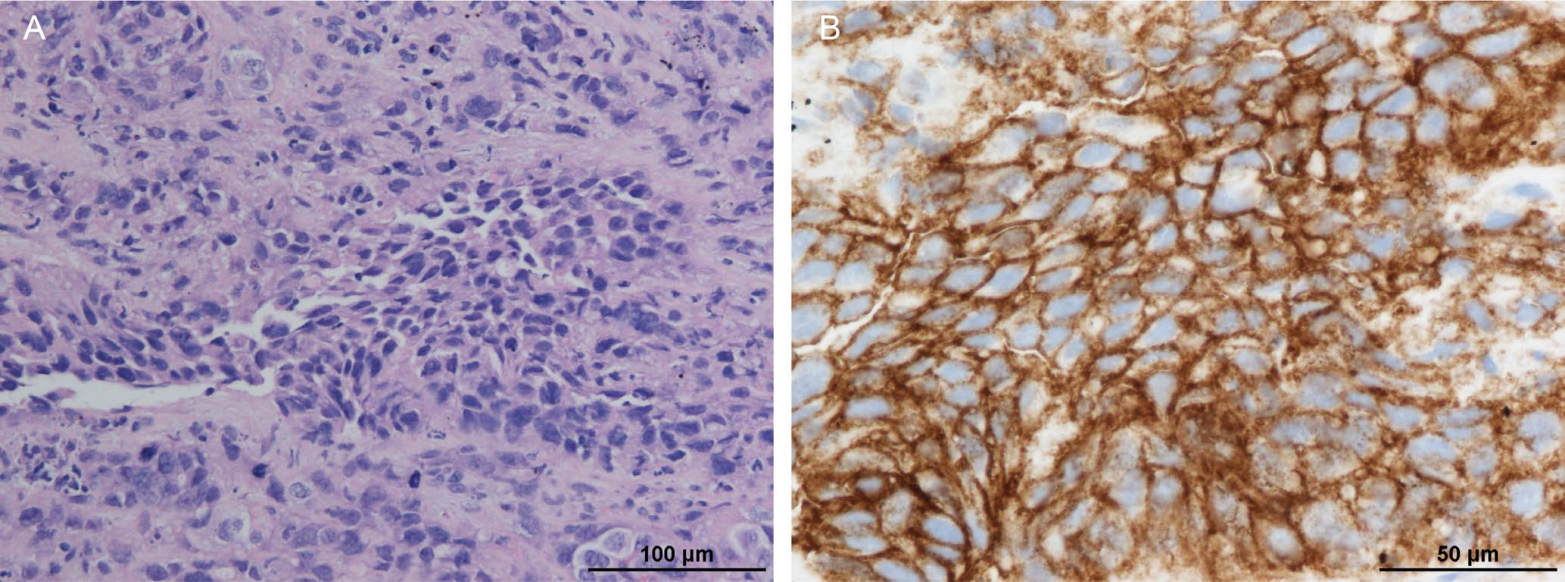
(A) Hematoxylin and eosin staining showing adenocarcinoma morphology (original magnification ×100; scale bar = 100 μm). (B) PD‐L1 immunohistochemistry using the 22C3 pharmDx assay demonstrating strong membranous staining in tumor cells (TPS 90%) (original magnification ×200; scale bar = 50 μm).

**FIGURE 2:**
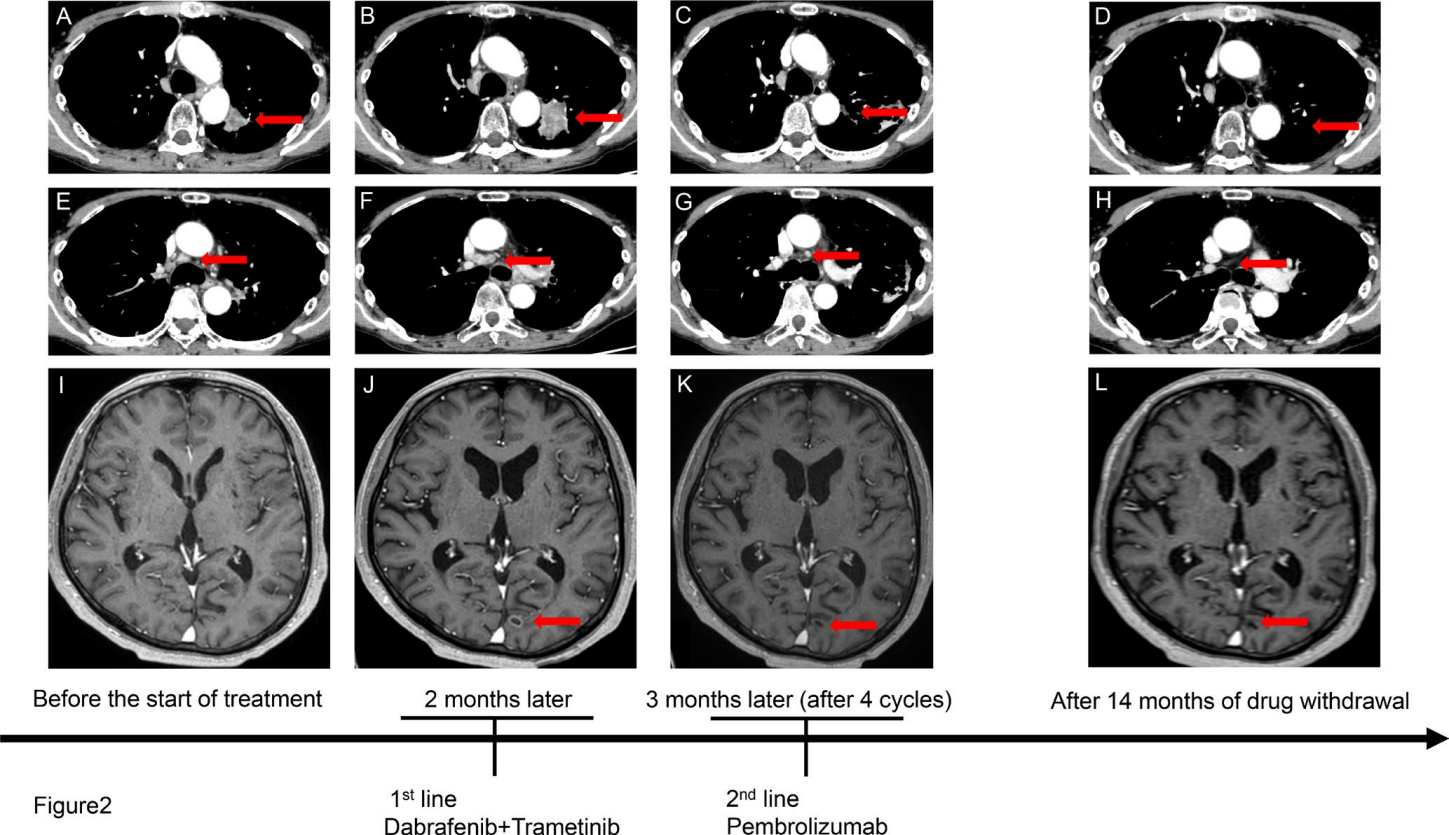
Representative CT and MRI images and treatment course over time. Images are shown before treatment initiation, 2 months and 3 months after treatment initiation, and 14 months after drug withdrawal. (A–D) Left upper lobe primary lung tumor (arrows). (E–H) Right lower paratracheal lymph node metastasis (arrows). (I–L) Left occipital lobe brain metastasis (arrows). CT, computed tomography; MRI, magnetic resonance imaging.

In February 2023, a man was clinically diagnosed with left upper lobe lung adenocarcinoma; the tumor was at stage cT2aN3M1c (BRA) IVB. Histopathological examination revealed tumor cells forming solid nests with enlarged nuclei and eosinophilic cytoplasm. Immunohistochemistry showed positivity for TTF‐1 and Napsin A and negativity for p40, supporting the diagnosis of lung adenocarcinoma. An Amoy Dx lung cancer multi‐gene polymerase chain reaction panel test was performed, and the BRAF V600E mutation was detected; PD‐L1 expression was also evaluated as positive (22C3 TPS: 90%, Figure 1). After radiotherapy for brain metastasis, the patient started first‐line treatment with dabrafenib and trametinib combination therapy in May 2023. One week after treatment initiation, therapy was interrupted for 7 days due to Grade 3 anorexia. Treatment was subsequently resumed on day 14 with a one‐level dose reduction (dabrafenib 300 mg to 200 mg daily; trametinib 2 mg to 1.5 mg daily). Two months after treatment initiation, imaging demonstrated enlargement of the primary tumor and mediastinal lymph nodes, along with the appearance of new brain metastases, findings consistent with progressive disease according to RECIST version 1.1 criteria (Figure 2). Pembrolizumab monotherapy was initiated in July 2023 as a second‐line treatment. After four cycles, the primary tumor, brain metastases, and mediastinal lymph nodes shrank (Figure 2). However, Grade 3 pneumonitis developed, and pembrolizumab was discontinued. One year and two months have passed since the last dose of pembrolizumab, and no progression of the primary disease has been observed.

## 3 Discussion

In BRAF V600E‐mutant NSCLC, response rates to BRAF/MEK inhibition are reportedly around 60%, but acquired resistance inevitably develops. In the present case, radiological progression was observed at the first evaluation despite temporary treatment interruption and subsequent dose reduction due to Grade 3 anorexia. The dose reduction was performed as a one‐level adjustment, similar to that reported in the phase II study of BRAF V600E‐mutant stage IV NSCLC, in which approximately 35% of patients required dose reductions [3]. Importantly, dose modification in that study did not appear to compromise anti‐tumor efficacy. Although a potential impact of dose reduction cannot be entirely excluded, early progression has also been reported in patients receiving full‐dose therapy, suggesting that primary resistance rather than dose modification was the more likely explanation in this case. Re‐biopsy was not performed; therefore, the underlying molecular mechanism of resistance could not be determined.

Recent real‐world studies and retrospective analyses have further characterized treatment outcomes in BRAF‐mutant NSCLC [5, 6], although optimal sequencing between targeted therapy and immunotherapy remains uncertain. Unlike EGFR‐ or ALK‐mutant NSCLC, BRAF‐mutant tumors are more frequently associated with a smoking history and relatively higher PD‐L1 expression levels [7]. In the present case, the tumor demonstrated very high PD‐L1 expression (TPS 90%) and the patient had a smoking history, both of which may have contributed to the favorable response to pembrolizumab.

Although most mechanistic data derive from melanoma, interactions between BRAF inhibition and the tumor immune microenvironment have been suggested [8, 9, 10, 11]. Modulation of PD‐L1 expression and immune cell infiltration following MAPK pathway inhibition may influence subsequent sensitivity to immunotherapy [12]. While direct evidence in NSCLC remains limited, such a dynamic interplay between targeted therapy and tumor immunity may partly explain the durable response to ICI observed after early targeted therapy failure in this case.

This case is unique in that it demonstrates early primary resistance to first‐line BRAF/MEK inhibition followed by a durable response to single‐agent pembrolizumab in a patient with very high PD‐L1 expression (TPS 90%). While BRAF V600E‐mutant NSCLC is generally treated with targeted therapy in the first‐line setting, accumulating evidence suggests that a subset of patients may derive substantial benefit from immune checkpoint inhibition. Our case contributes to this evolving understanding by highlighting the potential importance of PD‐L1 expression and clinical context when considering treatment sequencing in this molecular subtype.

## 4 Conclusion

In summary, this case highlights early resistance to BRAF/MEK inhibition and a durable response to ICI in BRAF V600E‐mutant NSCLC. High PD‐L1 expression and smoking history may help identify patients who could benefit from immunotherapy in sequential treatment strategies. Further prospective studies are warranted to clarify the optimal treatment sequencing in this molecular subtype.

## Author Contributions

**Yusuke Okayama:** writing – original draft, visualization. **Takaaki Tokito:** writing – review and editing. **Shizuka Shiraishi:** writing – review and editing. **Reiko Takaki:** writing – review and editing. **Shingo Tsuneyoshi:** writing – review and editing. **Hiroyoshi Yamada:** writing – review and editing. **Mamoru Nishiyama:** writing – review and editing. **Yoshiko Sueyasu:** writing – review and editing. **Tomoaki Hoshino:** writing – review and editing.

## Funding

The authors have nothing to report.

## Disclosure

The authors declare that this manuscript has not been published and is not under consideration elsewhere.

## Consent

Consent for publication was obtained from the patient.

## Conflicts of Interest

The authors declare no conflicts of interest.

## Data Availability

The data that support the findings of this study are available on request from the corresponding author. The data are not publicly available due to privacy or ethical restrictions.
